# Analytical Separated Neuro-Space Mapping Modeling Method of Power Transistor

**DOI:** 10.3390/mi14020426

**Published:** 2023-02-10

**Authors:** Xu Wang, Tingpeng Li, Shuxia Yan, Jian Wang

**Affiliations:** 1 State Key Laboratory of Complex Electromagnetic Environmental Effects on Electronics and Information System, Luoyang 471003, China; 2 School of Electronics and Information Engineering, Tiangong University, Tianjin 300387, China; 3 School of Microelectronics, Tianjin University, Tianjin 300072, China

**Keywords:** power transistor, modeling, neuro-space mapping, optimization method

## Abstract

An analytically separated neuro-space mapping (Neuro-SM) model of power transistors is proposed in this paper. Two separated mapping networks are introduced into the new model to improve the characteristics of the DC and AC, avoiding interference of the internal parameters in neural networks. Novel analytical formulations are derived to develop effective combinations between the mapping networks and the coarse model. In addition, an advanced training approach with simple sensitivity analysis expressions is proposed to accelerate the optimization process. The flexible transformation of terminal signals in the proposed model allows existing models to exceed their current capabilities, addressing accuracy limitations. The modeling experiment for the measurement data of laterally diffused metal-oxide-semiconductor transistors demonstrates that the novel method accurately represents the characteristics of the DC and AC of transistors with a simple structure and efficient training process.

## 1. Introduction

Power transistors are essential components in the microwave circuit system [1,2]. Therefore, high-precision transistor models play significant roles in system design [3,4,5]. Conventional modeling approaches, such as equivalent circuit models and empirical formula models, require slow trial-and-error processes. Due to the lack of freedom, traditional models often fail to meet the accuracy requirements. The electromagnetic (EM)-based design method is essential for accurate modeling. However, the EM model requires more information about device structure and material. In addition, expensive computing costs in EM simulation reduce transistor design efficiency [6,7,8,9]. Data-driven modeling methods with good flexibility and generality are applied to transistors, which eliminate the need for underlying device physics and laborious equation development [10,11,12]. The authors of [13] proposed a fully adaptive regression model (FARM), where processing functions and network components are obtained by the tree Parzen estimator automatically.

Recently, neuro-space mapping (Neuro-SM) has become an essential alternative to conventional modeling approaches in the microwave domain [14,15,16]. This method combines space mapping (SM) and an artificial neural network (ANN) as a Neuro-SM model. The measured or simulated data of the modeled devices are called fine models. Existing empirical formulas or equivalent circuits, which roughly represent the modeled device performance, are called coarse models. Mapping networks in Neuro-SM models make connections between coarse models and fine models. As a result, Neuro-SM methods could exceed the existing model’s accuracy limit and address the growing computational challenges in EM simulation. Neuro-SM modeling methods have been applied in many microwave device modeling fields [17,18,19]. The first Neuro-SM modeling technique, modifying the behavior of existing device models with novel formulations of space mapping, is presented in [20]. The trained model can accurately match device data and be applied to large signal circuit simulations. Compared with the circuit-based Neuro-SM method, an analytical Neuro-SM method with high computational efficiency is proposed in [21]. In [22], a dynamic neural network is introduced into the Neuro-SM model for microwave devices. The dynamic modeling technique makes up for non-quasi-static effects and any capacitive effects while retaining the response of static Neuro-SM. Two mapping networks are used to modify the current and voltage signals from the existing device model to the fine data in large-signal simulations [23]. A small-signal knowledge-based modeling approach, which uses the input and output package modules to improve the small-signal characteristic of transistors, is proposed in [24].

These existing methods use the same optimized variables to improve the characteristics of the DC and AC. The values of variables in mapping networks affect each other, resulting in increased difficulty in the training process. When the response trend between fine data and coarse models is inconsistent, or the nonlinearity of the characteristics of the DC and AC is high, existing Neuro-SM modeling approaches fail to satisfy the accuracy. The nonlinearity of the mapping network is controlled by both DC and AC performance, so an enormous number of free variables and complex neural network structures are needed to match the DC and AC responses. Different from DC characteristic modeling, AC characteristic modeling is more difficult for some existing methods. Existing methods can easily match the fine data at low power but fail to meet the accuracy requirement at high power. In order to enhance the accuracy, parameters in the model need to be constantly corrected, which has massive computing resource and time costs. Therefore, existing modeling methods could not meet the requirement of high precision and high efficiency at the same time.

A separated Neuro-SM for power transistor model is addressed in this paper. We propose adding two mapping networks to the traditional model, making the model more flexible. The proposed technique, which changes the characteristics of the DC and AC separately, can achieve better accuracy with simpler mapping networks than existing Neuro-SM modeling methods. In addition, an automatic training method is proposed to achieve higher accuracy and significantly reduce the repetitive training process to improve the modeling efficiency. The applications of the measured power transistor model verify the advantages of the separated Neuro-SM approach.

## 2. Model and Methods

### 2.1. Structure of the Separated Neuro-SM Model

**Figure 1:**
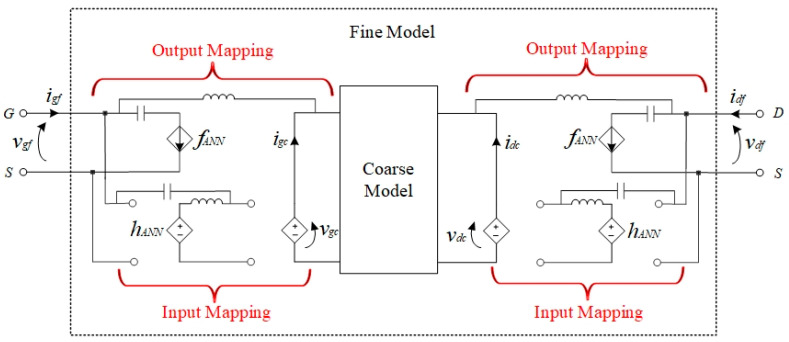
Circuit-based structure of the separated model.

Sometimes, the responses of existing models, such as empirical formulas or equivalent circuits, do not match those of the modeled device accurately, even if the parameters in the existing models are adjusted as much as possible. Mapping networks are introduced on the existing models to increase the degree of freedom. In this way, the existing model with low precision is taken as the coarse model, while the model combining the coarse model and mapping networks is called the Neuro-SM model. The goal of modeling is to obtain the same output response when the fine model and the Neuro-SM model operate with the same input parameters. To improve the modeling accuracy, we propose introducing inductors and capacitors into the traditional Neuro-SM structure, changing the characteristics of the DC and AC, respectively. The separated model’s circuit-based structure is given in Figure 1.

Here, the drain and gate of the transistor are denoted by subscripts *d* and g, respectively. Let subscripts *f* and *c* represent the fine model and the coarse model, respectively. In the proposed model, the input signals are the fine model’s voltage information, namely $v_{f}={\left[v_{gf},v_{df}\right]}^{T}$. $v_{gf}$ and $v_{df}$ represent the gate and drain voltage information of the transistor, respectively. Instead of directly acting on the coarse model, the two voltage signals are divided into DC and AC signals by inductors and capacitors, respectively.

The DC component is changed by the input mapping network, while the AC signal is not affected by the mapping relationship. In this way, the proposed model can improve the DC characteristic and keep the AC characteristic unchanged. Then, the input voltage information of the fine model is transferred to the coarse model defined as $v_{c}={\left[v_{gc},v_{dc}\right]}^{T}$. The coarse model’s output currents $i_{c}={\left[i_{gc},i_{dc}\right]}^{T}$ are obtained with the input voltage $v_{c}$. In this structure, instead of changing by the output neural network, $i_{c}$ is separated into two components. The DC component can pass directly without adjusting, but the AC component must be changed due to the inductance. The output mapping obtains a better AC characteristic without changing the DC characteristic. The response of the Neuro-SM model with two separated mapping networks can match the fine model output signals $i_{f}={\left[i_{gf},i_{df}\right]}^{T}$. This proposed method can improve the characteristics of the AC and DC and represent the device characteristics well with simple mapping relationships and a few optimization variables.

### 2.2. Analytical Formulation for Neuro-SM Method

New analytical formulations for the separated Neuro-SM model are proposed to represent the mapping mechanisms between the input and output signals. Instead of Kirchhoff equations and controlled sources, the model established by the analytical formulas can be trained and tested in one program, which speeds up the operation process. We achieve the analytical formulations within the environment of DC and AC cases.

#### 2.2.1. Analytical DC Signal Expression

**Figure 2:**
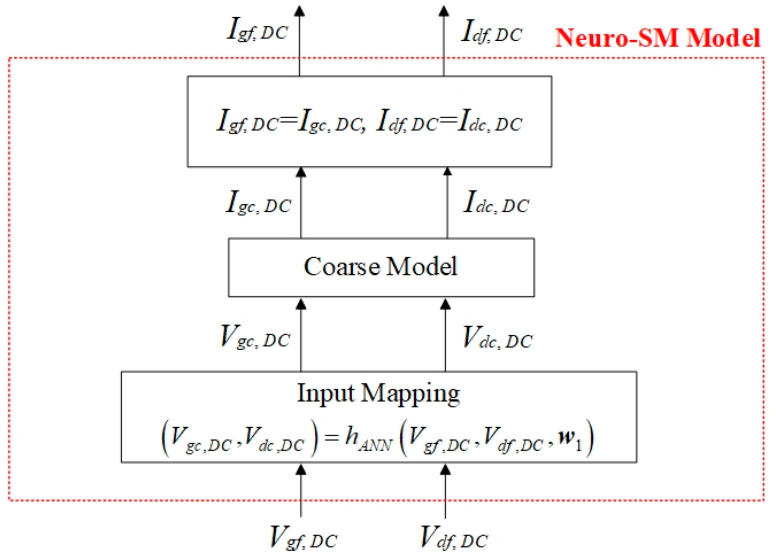
Schematic of the analytical DC model.

The DC signals of the transistor are supplied by the DC offset source, which directly affects the DC characteristic of the model. If the DC characteristic of the coarse model does not match the fine data well, the input mapping network is added to the input ports of the coarse model. Figure 2 shows the schematic of the analytical DC model.

In Figure 2, the fine input signals $V_{f,DC}$ are operated in the input mapping network instead of the coarse model. The DC response of the coarse model obtained with the coarse DC voltage signals $V_{c,DC}$ matches that of the fine model, i.e., $I_{f,DC}=I_{c,DC}$. The mapping network $h_{ANN}$ represents the nonlinear relationship between coarse signals $V_{c,DC}$ and fine signals $V_{f,DC}$. The function is

(1) $$\left(V_{gc,DC},V_{dc,DC}\right)=h_{ANN}\left(V_{gf,DC},V_{df,DC},w_{1}\right)$$

where $h_{ANN}$ denotes the multilayer perceptron network [6]. $w_{1}$ is the internal weights in $h_{ANN}$, which can be optimized in the training process. The activation function in $h_{ANN}$ is a sigmoid function, which is smooth and the derivative of which is easy to take. When the difference in the DC characteristic between the coarse model and the fine data is large, a complex network such as deep learning can be taken as the mapping network.

#### 2.2.2. Analytical AC Signal Expression

**Figure 3:**
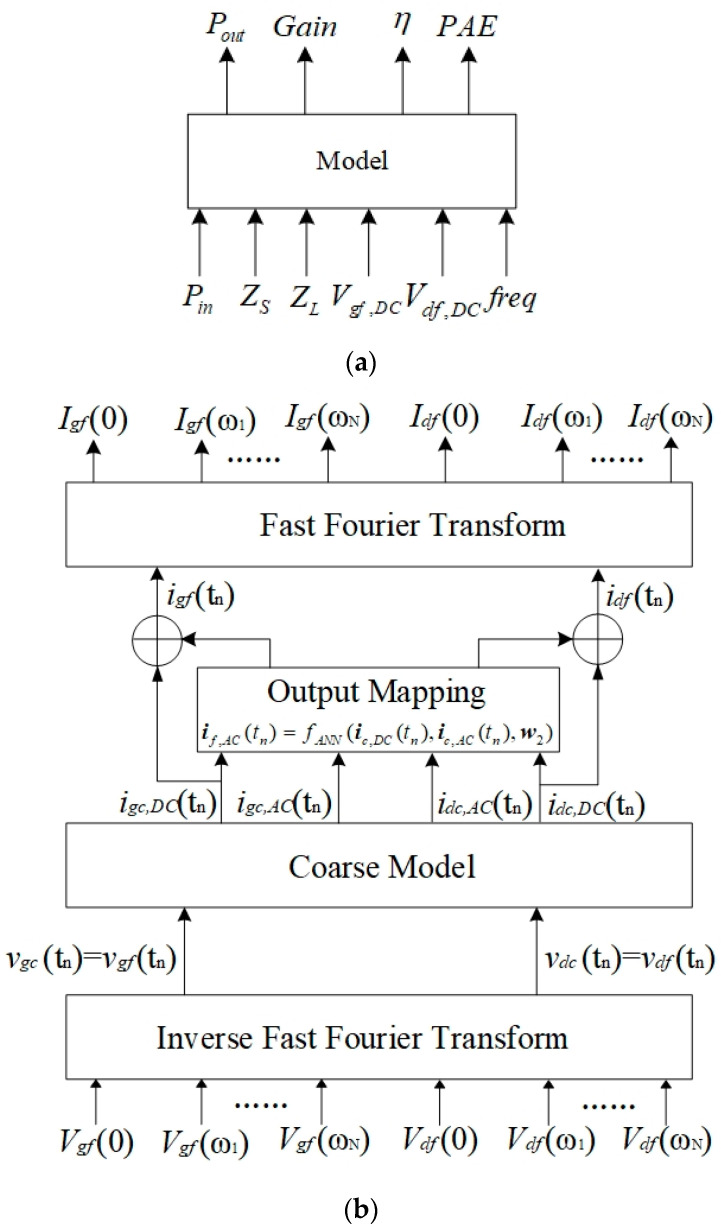
Schematic of the analytical AC model. (**a**) The input and output signals of the AC model. (**b**) The detailed process of the AC model.

The AC signals of the transistor mainly contribute to the large signal response. By adjusting the AC signal, the power response of the model can meet the precision requirements. If the AC characteristic of the coarse model does not match the fine data well, the output mapping network is added to the output ports of the coarse model. The output mapping network can improve the AC characteristic of the coarse model without affecting the DC characteristic of the coarse model. The schematic of the analytical AC model is given in Figure 3. The terminal signals of the new Neuro-SM model are shown in Figure 3a. The input signals of the AC model are bias voltages $V_{gf,DC}$ and $V_{df,DC}$, load impedance $Z_{L}$, source impedance $Z_{S}$, input power $P_{in}$ and frequency freq. The output signals of the model are output power $P_{out}$, power-added efficiency PAE, gain Gain and power efficiency η. Figure 3b shows the detailed process of the AC model, which includes the inverse fast Fourier transform module, coarse model, output mapping network and fast Fourier transform module. The accurate outputs of the AC model, which are $P_{out}$, Gain, η and PAE, can be calculated with the mapping currents and the excitation signals. The AC characteristic of the device is represented by the nonlinear relationship between the input and output signals of the AC model. An appropriate coarse model can provide a good foundation for AC characteristic modeling.

The proposed AC model is operated in harmonic balance (HB) simulation to demonstrate AC characteristics. HB simulation operates the frequency domain information of the terminal signals of the device, while the neural mapping network handles the terminal signals in the time domain. The output mapping network maps the time domain information of the current signals from the coarse model to the fine model. In Figure 3b, inverse fast Fourier transform (IFFT) converts input signals of the fine model to the time domain, and fast Fourier transform (FFT) converts output signals of the output mapping to the frequency domain. $V_{gf}(\omega_{k})$ and $V_{df}(\omega_{k})$ represent the harmonic voltages generated by the fine model at the harmonic frequency $\omega_{k}$. $I_{gf}(\omega_{k})$ and $I_{df}(\omega_{k})$ represent the harmonic current of the fine model. The index of the harmonic frequency is denoted by the subscript *k*, where k=0,1,2,……,N and N is the maximum harmonic number. $v_{c}(t_{n})$ represents input signals of the coarse model, which are equivalent to the signal of the fine model $v_{f}(t_{n})$. The DC and AC components of the coarse output signals are named $i_{c,DC}(t_{n})$ and $i_{c,AC}(t_{n})$, respectively. The AC current $i_{f,AC}(t_{n})$ is obtained by the output mapping network, and it can be expressed as follows:

(2) $$i_{f,AC}(t_{n})=f_{ANN}(i_{c,DC}(t_{n}),i_{c,AC}(t_{n}),w_{2})$$

where $f_{ANN}$ denotes the multilayer perceptron network [6]. $w_{2}$ is the internal weights in $f_{ANN}$, which can be optimized in the training process. The activation function in $f_{ANN}$ is a sigmoid function, which is smooth and the derivative of which is easy to take. When the difference in the AC characteristic between the coarse model and the fine data is large, a complex network such as deep learning can be taken as the mapping network.

Let ℱ() denote the FFT calculation in HB simulation, and then $I_{f}(\omega_{k})$ in the form of harmonics of the AC model is expressed as follows:

(3) $$I_{f}(\omega_{k})=ℱ(i_{f}(t_{n}))=\frac{1}{N_{T}}\sum_{n=0}^{N_{T}-1}\left[i_{c,DC}(t_{n})+f_{ANN}(i_{c,DC}(t_{n}),i_{c,AC}(t_{n}),w_{2})]\cdot W_{N_{T}}(n,k\right)$$

where n represents the sampling time point and $N_{T}$ is the maximum number of time points, i.e., $n=0,1,2,\ldots \ldots ,N_{T}$. $W_{N_{T}}(n,k)=e^{-j2\pi nk/N_{T}}$ is the Fourier coefficient for the *n*th sampling and the *k*th harmonic.

### 2.3. Sensitivity Analysis Expressions and Training Method

**Figure 4:**
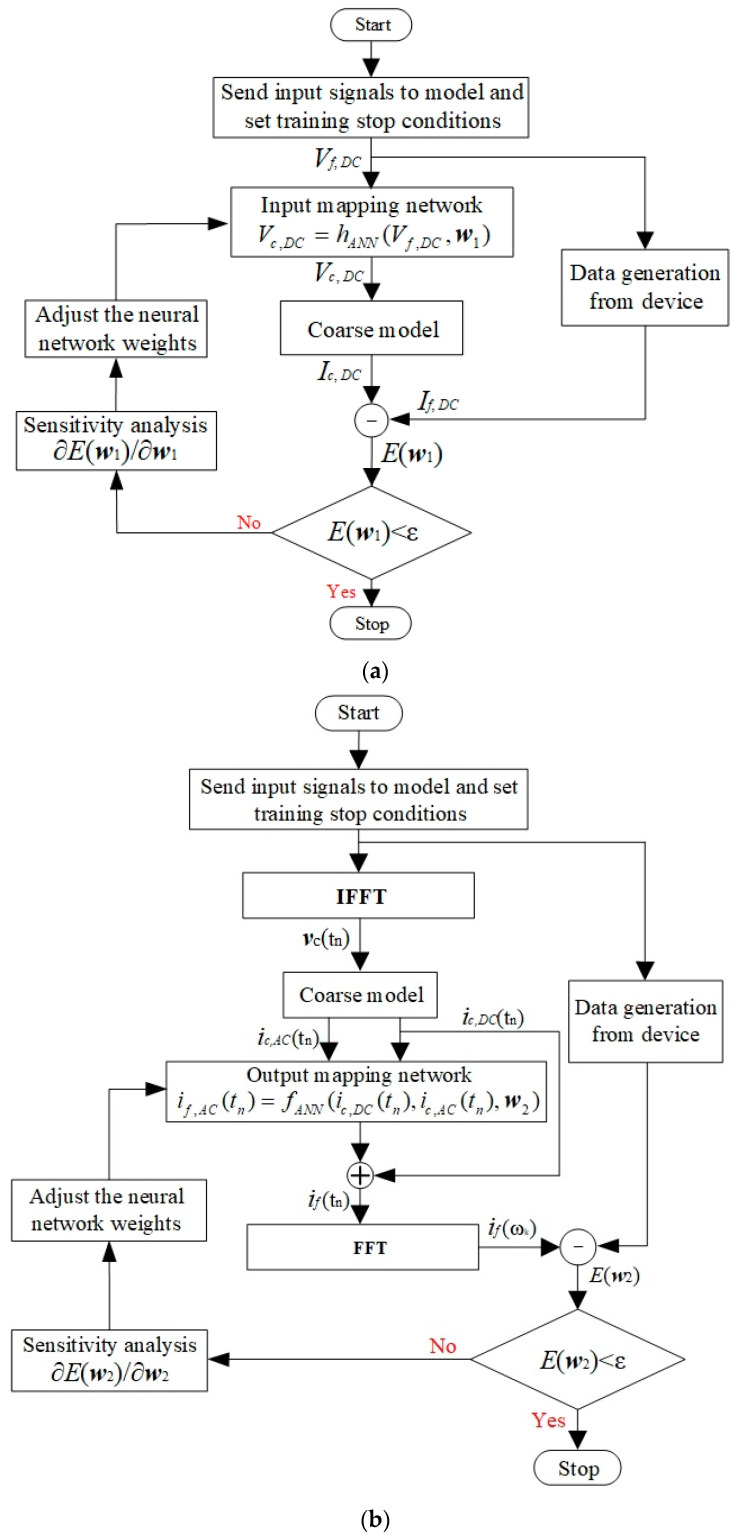
Advanced training process of the separated model. (**a**) DC model training. (**b**) AC model training.

An efficient training algorithm is an important part of transistor modeling, which determines the efficiency of the modeling process. This section introduces the new training method for the separated model. The training process of the DC characteristic model is shown in Figure 4a. In the DC model training, the input mapping neural network is trained to minimize the deference of DC between the new Neuro-SM model and the fine data. During training, the weights $w_{1}$ in the input mapping neural networks are optimized with the gradient information from the sensitivity analysis. We set $V_{c,DC}=V_{f,DC}$ to establish the input unit network, which prevents the training error from increasing. Meanwhile, the parameter $w_{1}$ in $h_{ANN}$ is changed in the training process, decreasing the errors between the data and the model conspicuously. When the training error between the data and the model meets the user-defined threshold *ε*, the DC training stage finishes. The training error reflects the learning ability of the developed model, and the test error is used to check the prediction ability of the developed model. When the training error is small and the test error is large, we can add more training data or reduce the hidden neurons of the mapping network. When both training error and test error are reduced to the threshold, the model can represent the modeled device well.

The training process of the AC characteristic model is shown in Figure 4b. The training data of the AC model are the harmonics of voltage signals and current signals. In the AC model training, the output mapping network is trained to minimize the gap between the harmonic balance response of the new model and the device data. The weight $w_{2}$ is adjusted with the gradient information. Set $i_{f,AC}(t_{n})=i_{c,AC}(t_{n})$ to obtain a suitable initial value of $w_{2}$ establishing the output unit network. Then, the weight parameter $w_{2}$ is optimized to reduce the difference between the data and the model output. The unit mapping networks maintain the error of the overall model while introducing new mappings. After training, the developed model is tested with the test data, which are never used in the training process. If the test error meets the accuracy requirement, the trained model is used instead of devices in large-scale circuits.

In the DC characteristic training method, the first-order derivative $\partial I_{f}/\partial w_{1,i}$ is required to speed up the training process. The parameter $w_{1}$ is the optimization variable. The sensitivity analysis expression of the DC model can be expressed as follows:

(4) $$\frac{\partial I_{f}}{\partial w_{1,i}}={\left(\frac{\partial I_{{}_{f}}^{T}}{\partial V_{c,DC}}\right)}^{T}\cdot \frac{\partial V_{c,DC}}{\partial w_{1,i}}=G_{c}\cdot \frac{\partial h_{ANN}(V_{f,DC},w_{1})}{\partial w_{1,i}}$$

where $G_{c}$ denotes the conductance matrix of the coarse DC characteristic, while $\partial h_{ANN}(V_{f,DC},w_{1})/\partial w_{1,i}$ denotes the first-order derivative calculated by the multilayer perceptron network [6].

In the AC characteristic training method, the first-order derivative $\partial I_{f}(\omega_{k})/\partial w_{2,i}$ provides the right direction for the next iteration, which can speed up the training process. The parameter $w_{2}$ is the optimization variable. The sensitivity analysis expression of this model can be expressed by the equation as follows:

(5) $$\frac{\partial I_{f}(\omega_{k})}{\partial w_{2,i}}=\frac{1}{N_{T}}\sum_{n=0}^{N_{T}-1}\frac{\partial f_{ANN}(i_{c,DC}(t_{n}),i_{c,AC}(t_{n}),w_{2})}{\partial w_{2,i}}\cdot e^{-j\cdot 2\pi \cdot nk/N_{T}}$$

The $l_{2}$ error represents the training error and test error between the separated model and the fine data, and the expression of the $l_{2}$ error is represented as follows:

(6) $$E(w)={\left[\frac{1}{N_{q}N_{p}}\sum_{q=1}^{N_{q}}{\sum_{p=1}^{N_{p}}\left|\frac{Y_{q}^{p}(w)-Y_{qD}^{p}}{{\left|Y_{D}^{p}\right|}_{\max}}\right|}^{2}\right]}^{{}^{\frac{1}{2}}}$$

where $Y_{qD}^{p}$ and $Y_{q}^{p}(.)$ are the DC or AC response of the fine data and the separated model; p and $N_{p}$ represent the index and the maximum number of the output signals, respectively, i.e., $p=1,2,\ldots \ldots ,N_{p}$; and q and $N_{q}$ represent the index and the maximum number of the training data, respectively, i.e., $q=1,2,\ldots \ldots ,N_{q}$.

## 3. Experiment and Discussions

The proposed method is applied to the laterally diffused metal-oxide-semiconductor transistor AFT18S230. In this example, the measured AFT18S230 data are the fine model [24]. The transistor AFT18S290 model in Advanced Design System (ADS), which has a similar performance to the fine model, is chosen as the coarse model. The coarse model with fixed parameters is used in DC and AC characteristic modeling. For DC characteristic modeling, the proposed model is trained at 180 different biases for 370 training iterations. Data for 50 biases different from the training data are used as the test data. For AC characteristic modeling, the device is operated with $Z_{L}=1.403-j3.748\;\Omega$, $Z_{S}=1.535-j4.232\;\Omega$, $V_{df}=28\;V$, $V_{gf}=2.75\;V$ and freq=1.805 GHz. The input power $P_{in}$ operates from 4.25 dBm to 40.25 dBm with a step of 2 dBm. The input powers 10.25 dBm, 20.25 dBm and 28.25 dBm are used as the test data, while other input powers are used as the training data. The proposed training process is operated in the software NeuroModelerPlus 2.0.

Before developing the separated Neuro-SM structure, a three-layer multilayer perceptron with 30 hidden neurons establishes the coarse model in NeuroModelerPlus. A model with the trained coarse model and two separated networks is developed after the development of the coarse model. The layers and the hidden neuron numbers of the mapping networks are determined after a lot of attempts. The higher the nonlinearity, the more layers and hidden neurons. The input mapping network has five. The training error combined with the DC response and AC response is 1.18%, while the test error for that is 1.24%. Both training error and test error are obtained by Equation (6). The trained model can process 10 sets of data in 0.005 s. The separated model with high accuracy and high efficiency can meet the requirements of electronics.

To verify the advantages of the separated model, we use two existing modeling methods. The traditional Neuro-SM model adding mapping networks at the coarse model’s input port in [21] and the Neuro-SM model improving both the current and voltage signals in [23] are called existing model 1 and existing model 2, respectively. The errors and the hidden neurons used in models are shown in Table 1. The test errors between the fine data and three Neuro-SM models are less than 3%, while the test error between the fine data and the coarse model is 8.82%. In addition, hidden neurons used in the proposed model are less than those of the two existing Neuro-SM models. In other words, the proposed modeling method matches the fine model with a much simpler mapping relationship.

**Figure 5:**
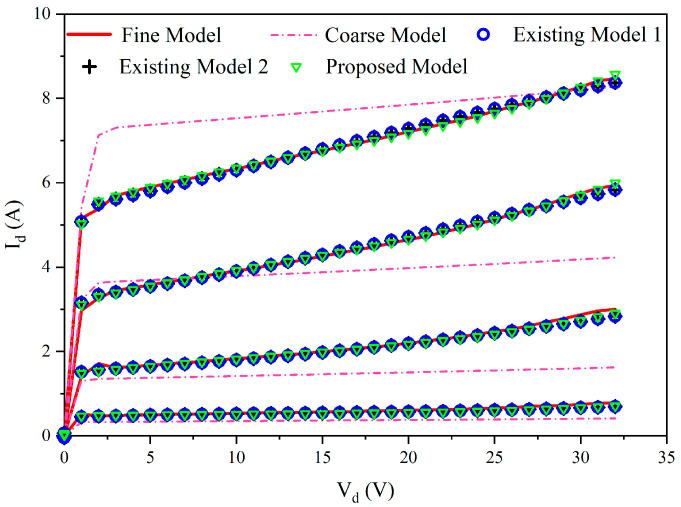
I–V comparison between the fine data and the models.

To further show the detailed results, we compared the DC responses of four models with the fine data at 230 bias. The errors of existing model 1, existing model 2 and the proposed model are 0.82%, 0.73% and 0.76%, respectively, while the error between the fine model and coarse model is 8.55%. It demonstrates that the separated model enhances the DC response of the coarse model, achieving the accuracy of the existing models. The I–V curves in Figure 5 show that the separated model perfectly represents the fine DC data.

**Figure 6:**
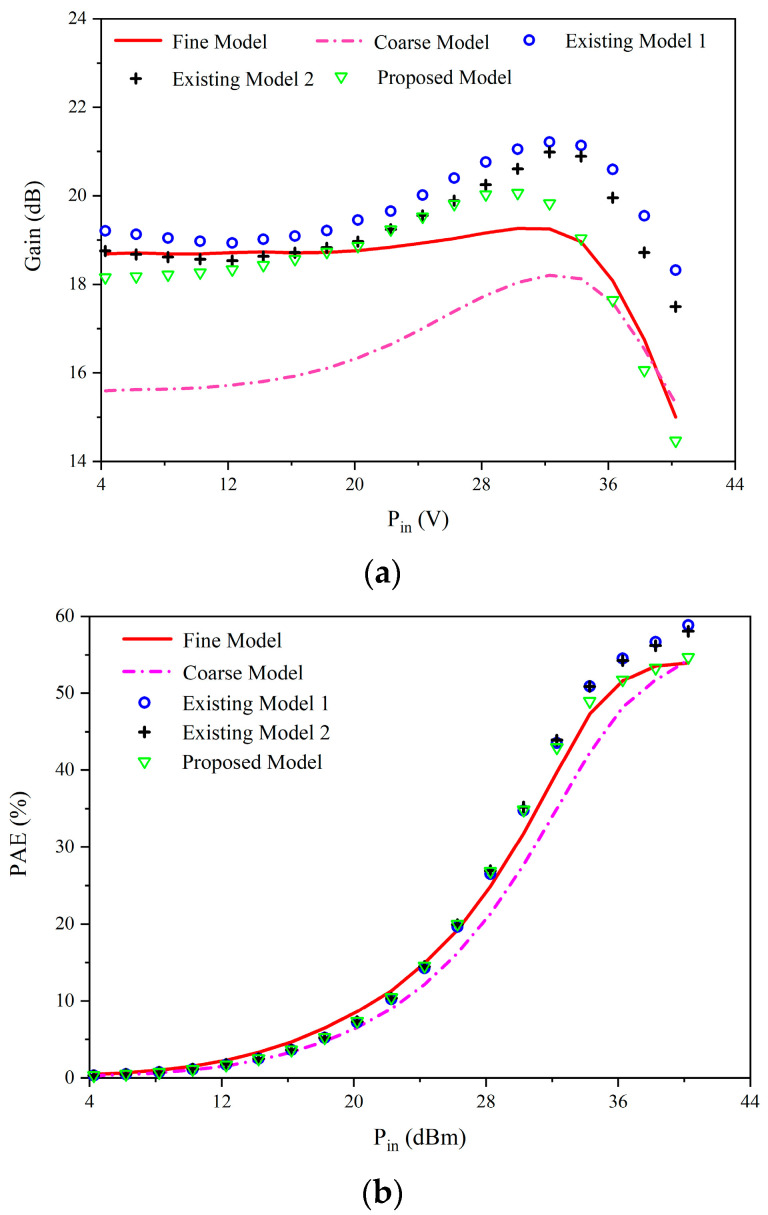
The parameters comparison between the models and the fine data. (**a**) Gain versus *P_in_*. (**b**) *PAE* versus *P_in_*.

For AC characteristic modeling, the proposed model has better accuracy than other existing Neuro-SM models. The AC error comparison of the coarse model, two existing Neuro-SM models and the proposed model is given in Table 2. Owing to the mapping networks with extra degrees of freedom, the separated model exceeds the capabilities of the coarse model. As a result, errors of the separated model are much smaller than those of existing model 1 and existing model 2. Two separated mapping networks are introduced into the proposed model to modify the DC signals and AC signals, avoiding variables’ interaction and reducing the optimization difficulty. Therefore, the proposed method has higher accuracy with fewer optimized variables. Figure 6 displays the gain and PAE comparison of the five models. The results verify that the separated model can match the measured data at all input powers, while existing model 1 and existing model 2 can only match the measured data at low powers. In a word, the proposed modeling method accurately shows the AC characteristics of the modeled device.

This paper derives sensitivity formulations of the separated model with mapping network weights. The proposed training algorithm with simpler sensitivity formulations can speed up existing training processes with perturbation sensitivity analysis. In contrast, the model developed by the circuit-based Neuro-SM method with three-section formulas at the coarse model output in [25] is used in this example. Simulated data from the ADS software are used to compare the training CPU time between the circuit-based model and the analytically separated Neuro-SM. The detailed results in Table 3 confirm that the analytically separated model has better efficiency.

## 4. Conclusions

In this paper, an effective model based on the separated Neuro-SM is proposed. Two mapping networks in the new model modify the characteristics of the DC and AC. A combination of the coarse model and the mapping structure is supplied by capacitors and inductors. The proposed training method’s analytical expressions and sensitivity analysis are derived to optimize appropriate weight values for mapping networks. Compared with the existing methods, the proposed model not only achieves good accuracy with less optimized variables but also speeds up the training process, improving the modeling efficiency. The measured power transistor example verifies the advantage of the separated model. In future work, larger-scale and more complex circuits with more measured data will be studied to verify the validity of the proposed method. In addition, various advanced modeling methods such as support vector regression machine (SVRM) and Gaussian process regression (GPR) will be applied for transistor modeling to further improve the modeling efficiency and accuracy.

## Figures and Tables

**Table 1:** Modeling results of four modeling methods.

Model Type	Hidden Neurons in Input Mapping	Hidden Neurons in Output Mapping	Training Error	Test Error
Coarse Model			8.77%	8.82%
Existing Model 1	30		2.83%	2.81%
Existing Model 2	15	15	2.21%	2.35%
Proposed Model	5	12	1.18%	1.21%

**Table 2:** HB simulation results of the four models.

Model Type	$P_{out}$	Gain	η	PAE
Coarse Model (%)	4.11	11.52	4.68	4.61
Existing Model 1 (%)	2.74	7.77	3.67	4.09
Existing Model 2 (%)	2.15	6.04	3.58	3.79
Proposed Model (%)	0.82	2.11	2.28	2.59

**Table 3:** Training time comparison of the models.

Data	Circuit-Based Model		Proposed Model	
	20 Hidden Neurons	30 Hidden Neurons	20 Hidden Neurons	30 Hidden Neurons
10 sets	12.7 s	13.8 s	1.5 s	1.9 s
30 sets	62.3 s	71.2 s	5.8 s	7.2 s
50 sets	79.7 s	84.4 s	6.7 s	10.1 s

## Data Availability

Not applicable.
